# THE ASSOCIATION OF AN ONLINE NATURE-BASED ASSISTED LIVING GROUP ACTIVITY PROGRAM AND RESIDENTS’ SOCIAL NETWORKS

**DOI:** 10.1093/geroni/igae098.0825

**Published:** 2024-12-31

**Authors:** Rebecca Mauldin, Jihye Baek, Keith Anderson, Megan Westmore, Anna Tulloh

**Affiliations:** The University of Texas at Arlington, Arlington, Texas, United States; Washington University in St. Louis, St. Louis, Missouri, United States; University of Mississippi, Oxford, Mississippi, United States; The University of Texas at Arlington, Arlington, Texas, United States; The University of Texas at Arlington, Arlington, Texas, United States

## Abstract

Assisted living (AL) residents may face a risk for social isolation related to high rates of disability, compromised social networks due to relocation, and segregation from their broader communities. Many AL communities offer opportunities for residents to connect with one another through shared meals, common areas, and group activities. Since COVID-19, online content is increasingly used in AL for economical and quarantine-resistant group activity content. We present findings from a mixed method study investigating changes in residents’ social networks associated with RASCALS: Reinforcing and Advancing Social Connectedness in Assisted Living. RASCALs is a group activity program that delivers and engages with content from online nature-focused livestreaming platforms. We presented Days at Dunrovin (livestreams, broadcasts, and videos from a ranch in Montana) twice a week for three months. Using a roster of resident names (N = 58), social networks (i.e., socializing partners and confidants) were assessed pre- and post-test. The number of socializing partners in the social networks in the assisted living buildings that received RASCALs programming increased during the quasi-experiment, while those in the comparison groups remained the same. There were no increases in the confidant networks of either group. In qualitative interviews, residents reported that RASCALs provided conversation topics and allowed residents to engage in increased small talk but did not foster close relationships with other residents. Findings indicate online nature-based group activities such as RASCALs may facilitate weak ties and socializing partners, relationships which have been associated with increased well-being, among assisted living residents.

